# Alcohol attention bias in 14-16 year old adolescents: an eye tracking study

**DOI:** 10.1007/s00213-020-05714-6

**Published:** 2020-12-03

**Authors:** Casey McGivern, David Curran, Donncha Hanna

**Affiliations:** School of Psychology, Queen’s University Belfast, David Keir Building, 18-30 Malone Road, Belfast, BT9 5BN Northern Ireland

**Keywords:** Alcohol, Adolescent, Attention bias

## Abstract

**Rationale:**

Theoretical models regarding the automaticity of attentional processes highlight a progression of attentional bias style from controlled to automatic in drinking populations as alcohol use progresses. Previous research has focused on older adolescent and adult drinking populations at later stages in their drinking career.

**Objectives:**

The aim of this study was to investigate alcohol attention bias in 14–16-year-old adolescent social drinkers and abstainers.

**Methods:**

Alcohol attention bias was measured in social drinking and abstaining groups in an eye-tracking paradigm. Questionnaires measured alcohol use, expectancies, exposure and socially desirable response styles.

**Results:**

Social drinkers fixated to alcohol stimuli more frequently and spent a larger proportion of their fixation time attending to alcohol stimuli compared to non-drinkers. Groups displayed differences in their style of attentional processing of alcohol-related information, with heavy drinkers fixating significantly longer to alcohol information across alcohol stimulus presentation and exhibiting a delayed disengagement style of alcohol attention bias that differentiated them from light drinking and abstaining peers. All social drinkers fixated significantly more than abstainers in the latter half of alcohol stimulus presentation.

**Conclusion:**

Alcohol attention bias was present in this adolescent sample. Drinking subgroups are defined from abstaining peers by unique features of their attentional bias that are controlled in nature. These findings are comparable to those in other adolescent and adult social drinking populations. The identification of specific attentional bias features according to drinking subpopulations has implications for our theoretical understanding of developing alcohol attention bias and problematic drinking behaviours, as well as at-risk identification and early intervention.

## Introduction

### Cognitive processes in addiction

In cognitive theories of addiction, automatic processes have been frequently highlighted and researched for their contribution to alcohol use disorder, specifically ‘alcohol attention bias’ (AAB). AAB is posited to contribute to alcohol misuse and dependence in theoretical literature such as the incentive sensitization theory of addiction (Robinson and Berridge 1993), which proposes that through the repeated consumption of alcohol, changes in the dopamine system occur, enhancing the pleasurable neurological effect of the substance. As exposure to the rewarding effects of alcohol is repeated, the same system begins to associate ‘wanting’ with alcohol-related information and subsequent cravings are produced. Once this association is established, alcohol information is incentivised, and preferential attention is assigned to it in an automatic and unconscious manner. These conditions facilitate triggers of craving and a ‘loss of control’ in alcohol use disorder that is characterised by relapse, even when an individual is consciously trying to abstain.

### The importance of adolescence

Most AAB research has focused on adult populations with longer established drinking patterns despite the key role assigned to early alcohol consumption behaviours in the formation of AAB and problematic alcohol use. McCusker’s (2006) automatic network theory of addictive behaviours illustrates the important role of early alcohol use in the development of the automatic cognitive processes implicated in addictive behaviours. Automatic responses in the attentional system are proposed to begin even before the commencement of alcohol consumption (likely in childhood), in the ‘pre-exposure’ phase. Here, largely positive alcohol-outcome associations (or expectancies) begin to be reinforced for the child through observational learning. These same outcome associations have been shown to signal increased levels of alcohol use in both adults and adolescents (see Young et al. 2006; McKay et al. 2011). During the second ‘exposure’ phase, a young person (usually in adolescence) begins to experiment with alcohol and alcohol-outcome associations are directly experienced, reinforced and repeated. As they are repeated, these expectancies can become associated with and activated by other internal- and external-related cues (e.g. alleviation of anxiety or social occasions) and a more automatic retrieval of positive associations and attention to alcohol-related stimuli develops. In the final ‘stereotypy’ phase (usually in adulthood), behaviourally repeated and signalled positive alcohol-outcome associations form responses in the autonomic system (signalled cravings), attentional system (automatic orientation to alcohol-related information is the environment) and propositional system (held attitudes such as ‘alcohol will relax me’) which can come to operate outside of conscious awareness. The automatic and biassed responses of each of these systems thus contribute to the behavioural impulse that characterises the ‘loss of control’ in problematic addictive behaviours, making it difficult for the individual to control their use or abstain.

The examination of AAB in adolescence is likely to offer unique insights into the developmental trajectory of this automatic cognitive bias found in problem drinking populations. In Northern Ireland, most individuals report first trying alcohol at age 12 (NISRA 2010), while 50% of Northern Ireland’s 15–16-year olds describe themselves as regular drinkers (Dempster et al. 2006).

### Alcohol attention bias

AAB is an observable, behavioural manifestation of these underlying and unconscious processes in addictive behaviour. Research examining the presence of AAB in alcohol-dependent adults (Lusher et al. 2004), and adult social drinkers (Miller and Fillmore 2011), with greater alcohol consumption associated with a more marked alcohol attentional bias (e.g. Schoenmakers et al. 2008; Fadardi and Cox 2009). Some studies have failed to replicate this differential finding (Field et al. 2007a), while others have highlighted that there are a number of other factors which can influence AAB in all populations of drinkers. Existing literature has provided rich accounts of the formation of AAB, however the evidence base is still developing. Research has largely focused on adult populations of alcohol-dependent and social drinking participants who likely have longer established drinking patterns and resulting AAB presentations.

The presence and pattern of attentional bias reported in the experimental literature has been identified primarily through reaction time tasks such as visual probe and Stroop methodology. These tasks infer AAB from varying reaction times to alcohol-related information (e.g. naming the colour of alcohol-related words more slowly than neutral words in a modified Stroop task or reacting more quickly to a symbol replacing alcohol-related stimuli in a dot-probe paradigm) but have been unable to identify more specific features of attentional processes in AAB. This is particularly important, as factors such as differences in drinking behaviour have been demonstrated to alter the features of AAB in adult drinkers. For example, alcohol-dependent adults have been shown to quickly and ‘automatically’ orient (within 50–100 ms during presentation; Noël et al. 2006) to alcohol-related information, while adult ‘heavy’ social drinkers have been shown to demonstrate alcohol AB on a later, more controlled level of attentional processing (within 500–2000 ms during presentation; Noël et al. 2006; Sacrey et al. 2013), and abstainers and light drinkers have demonstrated avoidant responses to alcohol-related information (Noël et al. 2006; Field et al. 2004). These findings paired with theory such as Robinson and Berridge (1993) suggest that AAB develops along a continuum from controlled to automatic as alcohol drinking patterns begin to become more established. A more sensitive and detailed measure of attentional responses to alcohol-related information has potential utility in aiding a more nuanced understanding of subgroup differences in the attentional processing of alcohol stimuli, potentially offering further insights into the development of AAB.

### AAB in adolescence

Field et al. (2007a), in comparing heavy and light drinking adolescents demonstrated the presence of AAB, while Zetteler et al. (2006) evidenced AAB in ‘at risk’ adolescents who had an alcohol-dependent parent. Both studies employed a Stroop paradigm to investigate AAB. While these studies were key in establishing and confirming the presence of AAB in adolescent populations (via reaction time tasks), they were unable to provide further insights into the development of AAB afforded by newer technologies.

Recent eye-tracking research by McAteer et al. (2015) has demonstrated AAB in 16–18-year-old adolescent social drinkers when compared to abstainers. AAB emerged in the later stages of stimuli presentation (1250–2500 ms), suggesting that the AAB in the sample was controlled in nature, as opposed to an automatic orienting to alcohol stimuli. As has been demonstrated in adults, the amount of alcohol consumed by the social drinkers, as well as positive alcohol outcome expectancies was positively associated to AAB. Later investigations by the same group of researchers (McAteer et al. 2018) found that the strength of AAB differed significantly between young adults and adolescents, providing a compelling support for the idea of the development of AAB occurring along a continuum defined by drinking experience. The current study utilised similar methodology using a younger age group with the aim of contributing to a more detailed understanding of AAB and its development.

Eye-tracking studies of AAB in adolescents are generally limited and no known study has used this methodology with this age group (14–16 year olds) to study AAB to date. By employing an eye-tracking paradigm, this investigation will add detail to the knowledge base regarding the features of AAB in adolescent social drinkers and abstainers. The features of the full attentional trajectory for alcohol-related stimuli will be investigated, as well as alcohol consumption and propositional relationships.

### The current study

The current study will investigate whether an AAB is present in this sample of heavy and light social drinking adolescents. If an AAB is present, we aim to identify whether attention to alcohol stimuli differs between groups of adolescent social drinkers (heavy and light drinkers) and abstainers. Furthermore, an investigation of the processes underpinning AAB will be undertaken to assess whether the bias is ‘automatic’ or ‘controlled’ in nature by assessing early and late viewing periods as well as attentional vigilance, delayed disengagement and attentional maintenance patterns.

Considering recent research evidence, the authors expect that social drinking adolescents will display an AAB that will differentiate them from abstaining peers and a relationship between AAB and alcohol consumption, and alcohol outcome expectancies will be found. Considering the theoretical literature as well as the findings of McAteer et al. (2015) it is expected that any AAB will be ‘controlled’ in nature, as opposed to an automatic pattern typically seen in problematic drinking populations.

## Methods

### Participants

Sixty-five adolescents were recruited from two consecutive school years in a secondary school in Northern Ireland. Fifty-eight participants (31 females) were included in the final analyses. The mean age of participants was 15 years, 3 months (SD = 0.58). Informed consent was provided by all participants and from their parents/legally authorised representatives. Participants were organised into three groups according to their AUDIT score: abstainers (scores 0), light drinkers (scores 1–8) and heavy drinkers (> 8). The study was approved by the ethics committee of the school of Psychology, Queen’s University Belfast.

### Materials

#### Questionnaires

Participants completed a short form enquiring about general demographic information such as age, sex, history of head injury and the presence of any other neurological deficits such as ADHD. They also answered a single question regarding subjective alcohol exposure, probing ‘how often are you surrounded by friends/family who are consuming alcohol?’ Possible responses were as follows: ‘Often’, ‘sometimes’, ‘rarely’ or ‘never’.

The Alcohol Use Disorders Identification Test (AUDIT; Allen et al. 1997) is a brief screening tool to detect problematic alcohol use. The questionnaire measures alcohol consumption, symptoms of dependency and consequences of drinking behaviour.

The Alcohol Expectancy Questionnaire—Adolescent (AEQ-A; Brown et al. 1987) is a 100-item measure which investigates general attitudes and alcohol outcome expectancies in 12–19-year olds across seven domains: cognitive and motor impairment, increased arousal, relaxation and tension reduction, global positive changes, changes in social behaviour, improved cognition and motor ability and sexual enhancement.

The short form of Crandall et al.’s (1965) Children’s Social Desirability scale (Baxter et al. 2004) consists of 14 direct questions to which the respondent answers yes or no in order to detect a desirable mode of responding.

#### Attention bias

Attentional processing was measured using a table mounted, video-based RED eye tracker (SMI Inc.), and stimuli were presented on a 22-in monitor with infrared optics attached to the bottom of the screen. Eye saccades were recorded at 250 Hz.

Alcohol-related images of words, single objects and complex scenes were matched with neutral images of a similar complexity, colour and size. Words were matched for length, use frequency and syllables. The stimuli have been previously rated by adolescents as low in emotional valence and appropriately alcohol-related according to stimuli type (‘alcohol related’ for alcohol stimuli and ‘not alcohol related’ for neutral stimuli). Please see McAteer et al. (2015, 2018) for information on the development and validation of the stimuli set.

### Procedure

Participants were invited to take part in a study of ‘everyday attention’ and completed the free-viewing eye-tracking task first. Participants were instructed to observe as they would naturally view a computer screen for the stimuli presentation. Prior to experimental stimuli presentation, participants viewed ten practice trials of neutral stimuli pairs. In experimental trials, participants were presented with 60 alcohol vs neutral stimuli pairs of varying complexity (20 word pairs, 20 single object pairs and 20 complex image pairs). A total of 30 neutral vs neutral stimuli pairs were randomly assorted in the stimuli presentation. The eye-tracking task was broken down into 3 sections (30 image pairs each) and participants had a short break between each presentation set. Trials were counterbalanced to prevent any left gaze bias affecting results (McAteer et al. 2015). Stimuli pairs were presented for 2500 ms each with a fixation cross presented between each pair for 1000 ms to re-orient the participant for the next trial. Following the eye-tracking task, participants completed questionnaires measuring their alcohol consumption, explicit alcohol outcome expectancies, social desirability of responses, subjective vicarious exposure to alcohol and demographic information before debriefing.

### Data preparation

Fixations on ‘areas of interest’ (AOIs) were coded for experimental trials only (alcohol vs neutral stimuli). Fixations were classed as focused attention on one AOI for at least 100 ms (as suggested by Manor and Gordon 2003 for free-viewing tasks). When creating AOIs, a border of 1 cm was placed around each stimulus, and all fixations which fell within this border were taken as a fixation to the AOI. All other fixations were excluded from analyses.

Considering distinctions between automatic and controlled attention, four eye patterns were prepared for analysis (Bradley et al. 2016):

#### Measure of vigilance bias

Direction of initial fixation (frequency first fixation to alcohol or neutral stimuli) was extracted to assess for a vigilance bias in attention.

#### Measures of delayed disengagement bias

The duration of the first fixation on each stimuli type was used to measure patterns of delayed disengagement.

#### Measures of maintenance bias

The total number of fixations on each stimuli type (total fixation count) and the total duration of fixations on each stimuli type were extracted as measures of maintained attention.

In order to compare participant groups, the direction of the initial fixation (to alcohol or neutral stimuli), the duration of first fixation on each stimuli type and fixations to each AOI are expressed as mean percentages of overall initial fixations and fixation times to AOIs. Some participants could not be accurately calibrated to the eye-tracking measure, 7 participants with a mean calibration > 1^0^ (across *x* and *y* coordinates) were excluded from the study as calibrations above this threshold were deemed to lack accuracy. This was largely due to participants wearing dark rimmed eye glasses that prevented the accurate tracking of their eye movement. One participant reporting a diagnosis of attention deficit hyperactivity disorder (ADHD) was also excluded from the study.

## Results

### Group characteristics

As shown in Table 1 Heavy drinkers (*n* = 15) scored highest for alcohol consumption levels on the AUDIT, followed by light drinkers (*n* = 21) and, as expected the abstaining group (*n* = 22) scored lowest. While the majority of the sample reported having tasted alcohol, six abstainers reported never having tried alcohol. The heavy drinking group had the youngest mean age at first drink of alcohol, followed by light drinkers and abstainers. There were significant differences between the groups’ age upon first drink of alcohol (*F*(2,55) = 3.326; *p* = .043); post hoc analysis showed that light drinkers tried alcohol significantly later than abstainers, who had the youngest mean age of first drink. No significant differences in alcohol consumption levels were evident between males and females (*F*(2, 55) = 1.327; *p* = .274).

**Table 1:** Descriptive statistics for alcohol use, alcohol expectancy scores and social desirability scores for abstaining, light drinking and heavy drinking groups

	Total (*n*)	Mean age (standard deviation)	AUDIT mean score (standard deviation)	AUDIT Range (0–20)	AEQ-A total score (atandard deviation)	Social desirability score mean (standard deviation)	Social desirability range (0–14)	Mean age at first drink (standard deviation)	Mean subjective exposure score
Abstainers	22	15.14 (.56)	0 (0.00)	0	7.72 (9.76)	4.27 (3.35)	0–13	7.86 (5.45)	1.55 (.96)
Light drinkers	21	15.43 (.51)	3.71 (2.41)	1-8	14.71 (9.58)	4.14 (3.04)	0–13	11.05 (2.87)	2.19 (.75)
Heavy drinkers	15	15.53 (.64)	13.4 (4.37)	9-23	22.13 (9.09)	2.27 (2.05)	0–6	9.8 (3.01)	2.27 (.80)

#### Questionnaires

As would be expected, there was a significant main effect of group on alcohol consumption scores (*F*(2, 55) = 117.557; *p* < .001). There was also a significant main effect of group on AEQ-A total scores (*F*(2,55) = 10.289; *p* < .001). Post hoc analysis indicated that heavy drinkers scored significantly higher than abstainers (*p* < .001) on the AEQ-A. A significant main effect of group was found for reported exposure to others’ alcohol consumption (*F*(2, 55) = 4.376; *p* = .017) with post hoc analysis indicating that heavy (*p* = .042) and light drinkers (*p* = .047) are in the company of others who are consuming alcohol significantly more often than abstainers.

#### Correlation analyses

Alcohol use correlated negatively with the tendency to answer in a socially desirable manner (*r*(58) = − .291; *p* = .027) and correlated positively with exposure to others’ alcohol consumption (*r*(58) = .304; *p* = .020) and alcohol expectancy scores (*r*(58) = .452; *p* < .001). That is, as reported alcohol consumption levels increased, socially desirable responding styles decreased and reporting of being in the company of others who are drinking, as well as positive expectancies of alcohol use increased. Attention maintenance (mean total fixation time) to alcohol stimuli significantly positively correlated with alcohol consumption (*r*(58) = .433; *p* = .001) but not with AEQ-A total score (*r*(58) = .249; *p* = .060).

### Attention bias

#### Vigilance

The direction of initial fixation towards alcohol or neutral stimuli was used to explore vigilance bias in the data. Drinkers did not preferentially attend to alcohol stimuli first (*F*(2, 55) = 0.681; *p* = .510).

#### Delayed disengagement

The duration of the initial fixation to alcohol stimuli was assessed as an indicator of delayed disengagement bias. There was a main effect of group on initial fixation duration to alcohol stimuli (*F*(2, 55) = 4.07; *p* = .023). Post hoc analysis showed significant differences between heavy drinkers and abstainers in their length of first fixation to alcohol stimuli (*p* = .028).

#### Maintenance

**Fig. 1:**
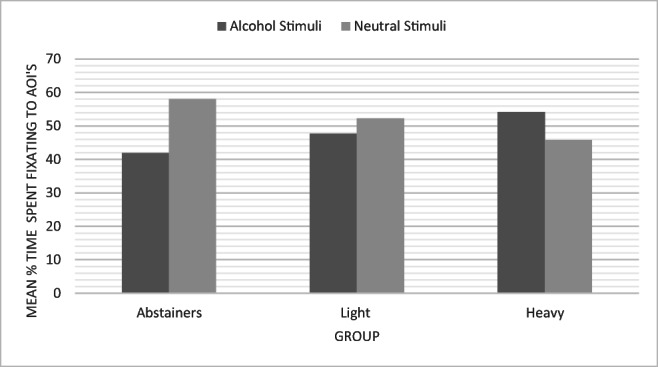
Bar chart displaying total fixation time to alcohol and neutral stimuli for abstaining, light drinking and heavy drinking groups

To explore a maintenance bias in attention to alcohol stimuli, mean total fixation time and number of fixations to alcohol and neutral stimuli were analysed. A significant main effect of the group was found for total fixation time (*F*(2, 55) = 9.44; *p* < .001), with post hoc analysis showing significant differences between heavy drinkers and abstainers (*p* < .001) and light drinkers and abstainers (*p* = .045) (see Table 2). Means show that heavy drinkers spent the largest proportion of viewing time fixated to alcohol stimuli (see Fig. 1).

**Table 2:** Mean percentage data for attention bias measures

	Total (*n*)	Vigilance bias	Delayed disengagement bias	Maintenance bias	
		First fixation to alcohol stimuli (standard deviation)	Mean first fixation length for alcohol stimuli (standard deviation)	Mean Total fixation time to alcohol stimuli (standard deviation)	Mean Total fixation count to alcohol stimuli (standard deviation)
Abstainers	22	47.14 (4.79)	45.11 (10.66)	40.58 (11.78)	43.11 (10.96)
Light drinkers	21	46.38 (5.68)	49.88 (4.31)	47.81 (5.52)	48.42 (3.82)
Heavy drinkers	15	48.40 (5.09)	52.03 (5.84)	54.13 (9.86)	52.92 (6.08)

A main effect of group was evident for total fixation count for alcohol stimuli (*F*(2, 55) = 7.274; *p* = .002). Post hoc analysis showed that heavy drinkers fixated to alcohol stimuli significantly more frequently than abstainers did (*p* = .001).

#### Early versus late attention

To examine whether alcohol attention bias was underpinned by early or late attentional processes, the stimuli presentation times were divided in two-halves(Laidlaw et al. 2012), and the mean fixation times for both were analysed. The first half of the stimuli presentation (0–1249 ms) was considered an early viewing period, while the second half (1250–2500 ms) was considered a measure of prolonged, late attention. Means are shown in Table 3. A significant main effect of group was found for fixation time to alcohol stimuli in both the first (*F*(2, 55) = 7.051; *p* = .002) and second half (*F*(2, 55) = 10.371; *p* < .001) of the stimuli presentation.

**Table 3:** Mean percentage of fixations to alcohol and neutral stimuli during first and second half of stimuli presentation

	Time of presentation (ms)	Abstainers (standard deviation)	Light drinkers (standard deviation)	Heavy drinkers (standard deviation)
Mean fixation time to alcohol stimuli	0–1249	46.60 (6.87)	48.90 (5.41)	54.80 (7.64)
	1250–2500	44.53 (4.92)	48.32 (3.65)	51.47 (5.33)
Mean fixation time to neutral stimuli	0–124	53.40 (6.87)	51.10 (5.41)	45.20 (7.64)
	1250–2500	55.47 (4.92)	51.68 (3.65)	48.53 (5.33)

Post hoc analysis showed that heavy drinkers looked significantly longer at alcohol stimuli than light drinkers (*p* = .032) and abstainers (*p* = .001) in the first half of the presentation. While in the second half, they looked significantly longer than only the abstainers (*p* < .001). In the second half of the presentation, light drinkers looked significantly longer at alcohol stimuli than abstainers (*p* = .028), but not in the first half. See Table 3 for means.

## Discussion

### Summary

Findings indicate that an alcohol AB was present in this adolescent sample. Social drinkers fixated to alcohol stimuli more frequently and spent a larger proportion of their fixation time attending to alcohol stimuli compared to non-drinkers. These findings lend support to previous work in older adolescent populations (McAteer et al. 2015). Groups displayed differences in their style of attentional processing of alcohol-related information, with heavy drinkers fixating significantly more towards alcohol information in early presentation times than light drinking and abstaining peers, and all social drinkers fixating significantly more than abstainers in the latter half of the stimulus presentation.

### Alcohol attention bias

#### Vigilance

No vigilance bias or automatic orienting was found in the current sample of adolescent social drinkers and abstainers as no immediate attentional preference was automatically allocated to alcohol stimuli when presented. This finding is in keeping with study findings such as that of Noël et al. (2006) that found a vigilance bias in alcohol-dependent populations, but not social drinking populations. Indeed, other studies have reinforced this idea in the literature that a vigilance bias is a feature of alcohol dependence and not social drinking populations, who tend to display attentional bias features beyond the immediate and initial presentation of alcohol stimuli (Vollstädt-Klein et al. 2009; Field et al. 2004; Stormark et al. 2000). Considering this finding and the existing literature, social drinking groups appear different from alcohol-dependent populations and may not have yet reached automaticity. This idea lends itself well to the notion of a continuum of alcohol attention bias ranging from controlled to automatic processes as discussed in previous theoretical models (McCusker 2006; Robinson and Berridge 1993) with varying qualitative features of attentional bias across drinking sub populations.

#### Delayed disengagement

A delayed disengagement bias differentiated heavy social drinking adolescents from their abstaining peers. That is, while heavy social drinkers did not automatically orient to alcohol stimuli more often than neutral stimuli, when they did look at alcohol stimuli first, they looked at it for significantly longer than abstaining peers. Considering AAB on a continuum, this is perhaps the first stage in which social drinking groups, specifically heavy drinkers, can begin to be differentiated in their attentional processing style according to alcohol consumption. This pattern of attentional bias perhaps suggests a difficulty disengaging one’s attention from alcohol stimuli once it is in focus in order to reassign attention elsewhere. This style has been referred to as ‘sticky’ in previous studies (McAteer et al. 2015; Sacrey et al. 2013; Hanania and Smith 2010) where it has been described as an emerging feature of selective attentional control.

#### Maintenance

Heavy social drinkers continue to differentiate from abstaining peers when considering a maintenance bias of attention for alcohol stimuli, particularly the frequency or ‘count’ of fixations. They look more frequently at alcohol stimuli than abstainers, again indicating a ‘sticky’ style of attentional processing once their attention is captured by alcohol-related stimuli. Other features of a maintenance bias in attentional processing begin to define all social drinking groups from abstainers when the total proportion of fixation time to alcohol stimuli is considered. All social drinkers spent significantly more time fixating to alcohol stimuli across the full stimuli presentation than abstaining peers. The differences in the maintenance bias findings for frequency of fixations vs total fixation time are interesting, as frequency only successfully differentiates heavy drinkers while total fixation time more successfully captures all drinking populations.

#### Early vs late attentional processes

Investigation of maintained attention bias tendencies in the first and latter half of the stimuli presentation indicated differences between heavy and light social drinkers. In the first half of the stimuli presentation, heavy social drinkers look longer than their light drinking and abstaining peers, whereas in the second half of the presentation, all social drinkers differed from abstainers. McAteer et al. (2015) found that only the latter half of the stimuli presentation differentiated between heavy drinkers and abstainers, with no significant differences for maintenance bias in the first half. The current findings suggest that social drinking groups may differ in terms of AAB features that they display, and that sensitivity of AAB measurement is important in such populations. There is also an indication that specific attentional features differentiate between drinkers according to their level of experience of and exposure to alcohol. This finding is in support of existing literature (Townshend and Duka 2001, 2007) that has previously defined heavy social drinkers from light social drinkers in longer stimuli presentation trials in visual probe tasks. Thus suggesting that a more controlled attentional bias defines social drinking groups generally, and that subgroups exhibit unique features according to alcohol consumption levels.

### An AAB continuum: developmental insights

**Fig. 2:**
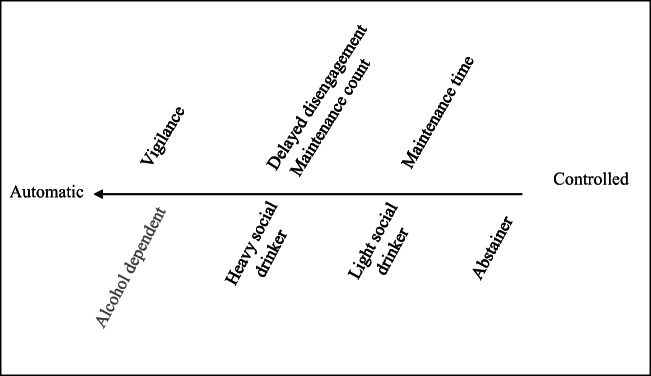
A visual conceptualisation of specific features of attentional bias for alcohol and drinking populations mapped to a continuum of controlled toautomatic processing

These findings would suggest that subtle differences in the strength and qualitative features of the adolescent social drinking groups’ attention bias were evident when compared to abstaining peers. Previous authors have proposed a continuum-based understanding of attentional bias from controlled to automatic processing (see McCusker 2006; Robinson and Berridge 1993). From a theoretical perspective, if on the ‘automatic’ end of the continuum vigilance for alcohol stimuli (looking at alcohol stimuli first) is a unique feature to alcohol dependence, moving towards the ‘controlled’ end of the continuum in this sample, a delayed disengagement bias (the duration of initial fixation to alcohol stimuli) and a maintenance frequency bias (the amount of times they looked at alcohol stimuli) differentiate heavy social drinking groups. This is followed by a maintenance time bias (the total time spent viewing alcohol stimuli), where all groups of social drinkers (light and heavy) can be defined, and finally by no attentional bias exhibited by the abstainers. Mapping these specific, observable features to the current theoretical understanding of alcohol attention bias can lend detail to our understanding of AAB development, a visual conceptualisation is provided in Fig. 2 below.

It is possible that increased experience of, and exposure to, alcohol will result in subtle changes in attentional processing style over time, of which the most automatic seems somewhat resistant to change. For example, Stormark et al. (1997) demonstrated that alcohol-dependent patients in treatment still exhibit an automatic vigilance bias for alcohol, despite abstention and active avoidance of alcohol-related stimuli later in stimuli presentations (Noël et al. 2006; Townshend and Duka 2001, 2007). This opens discussion regarding the role of attentional avoidance in drinking and abstaining populations, including where it may lie on a continuum of automatic to controlled alcohol attention bias. Abstainers in this study and others preferentially attend to neutral stimuli or actively avoid alcohol stimuli. This shared characteristic amongst these two groups indicates the importance of current levels of consumption as well as general alcohol experience when considering the full trajectory and style of attentional processing in the groups and warrants further investigation in the literature.

### Clinical implications, future research and limitations

This study supports previous findings and offers further insights to the collective understanding of alcohol attention bias in adolescent drinking populations. If vulnerable groups can be identified for early intervention, it may be possible to prevent a progression towards a more automatic style of attentional processing, and subsequent problematic drinking behaviour. Treatments seeking to alter and reduce attentional biases may be useful for vulnerable, heavy social drinking populations. Indeed, attention processing styles have been demonstrated as malleable in the literature, for example controlled attentional processes have successfully responded to training and manipulation techniques in problem and non-problem alcohol drinking populations (Fadardi and Cox 2008; Schoenmakers et al. 2010; Wiers et al. 2011) and other clinical presentations such as anxiety (MacLeod et al. 2002; Beard et al. 2012; Linetzky et al. 2015). Subsequent changes in attentional processing style as well as more favourable clinical outcomes have been noted in these populations, for instance following retraining, problem drinkers’ relapsed 13% less in the following year compared to controls (Eberl et al. 2013). Arguably, identifying and attempting to modify attention bias holds clinical merit that warrants further investigation in a wider range of drinking groups. The authors note that attentional retraining methods are at a relatively early stage of research and application, and findings have also questioned their generalisability (Field et al. 2007b). Study technology could also be utilised in a novel way in this area, or example eye-tracking technology may also serve as a useful intervention tool for future use in exposure therapies, implementing redirecting strategies and relapse prevention planning, as well as monitoring attention bias automaticity and its modification as treatment progresses.

Other attentional patterns from qualitatively unique groups currently underrepresented in the literature would also make a valuable contribution to our understanding of the development of problematic drinking behaviours and alcohol attentional bias. For example, by investigating alternative developmental pathways in adolescents’ relationship with alcohol and subsequent attentional biases in adolescent groups classed as ‘hidden harm’ who have an alcohol-dependent parent. As discussed, Zetteler et al. (2006) found AAB in a hidden harm adolescent sample in a modified Stroop task. However, eye-tracking methodology could capture more detailed attentional features and inform on the potential impact of largely negative vicariously experienced alcohol outcomes mentioned in McCusker’s (2006) automatic network theory prior to their own direct consumptive exposure to alcohol.

The use of a non-clinical sample in the current study of adolescent drinkers can limit the generalisability of the findings; however, it remains clinically important to capture these populations to inform our theoretical understanding, at-risk identification and early intervention strategies. Situating these findings along a certain developmental trajectory is not ideal when comparing with separate cross-sectional findings from drinking groups of different ages and consumption levels in the literature. Ideally, future research would combine clinical and non-clinical groups of varying ages and alcohol consumption levels for a more direct, detailed and longitudinal comparison.

## Conclusions

The current findings provide evidence that unique attentional bias features are related to alcohol consumption levels. AAB is present within this sample of social drinking adolescents. Drinking subgroups are defined from abstaining peers by unique features of their attentional bias that are controlled in their nature, with no vigilance bias evident. These findings are comparable to those in other adolescent and adult social drinking populations. The identification of specific attentional bias features in drinking subpopulations has implications for our theoretical understanding of developing alcohol attention bias and problematic drinking behaviours, as well as at-risk identification and early intervention.
